# Actual concepts in scaphocephaly (an experience of 98 cases)

**Published:** 2011-11-24

**Authors:** AV Ciurea, C Toader, C Mihalache

**Affiliations:** * "Bagdasar-Arseni" Emergency Hospital, Bucharest; ** National Neurology and Neurovascular Diseases Institute; *** "Sf. Andrei" Hospital, Neurosurgery Department, Galati

**Keywords:** Craniosynostoses, simple craniosynostoses, sagittal suture, scaphocephaly, 3D CT

## Abstract

Craniosynostoses are recognized as a group of birth defects that impair the skull structures by early closure of one or more sutures, causing an abnormal cranial shape. Among the "simple" craniosynostoses, (a single closed suture) the most common is scaphocephaly. The 3D CT scan is the most relevant and rapid diagnostic test. The authors present the personal experience of 98 scaphocephaly cases diagnosed and surgically treated in the Neurosurgical Department of "Bagdasar-Arseni" Emergency Hospital during a period of 10 years (2000 – 2009). The procedure of choice was the Stein & Schut (1977) extensive craniotomy that removes the early closed suture. There were no post-operatory death cases and no abnormally closed sutures. The routine use of the craniotome facilitates the lateral osteotomy that allows a normal brain growth and a normal symmetrical skull shape development. The authors advocate for early surgery during the first 6 months of life.

## Introduction

Craniosynostoses represent a heterogeneous group of nosographic entities characterized by the premature fusion of one or more sutures of the skull that result in a complex craniofacial malformation.

The phenomenon of early closure of one or more cranial sutures results in a variety of functional and morphological alterations of the craniofacial development and in different degrees of cranio-cerebral volumetric disproportions.

Virchow R., (1859) defines the craniosynostoses as early closure of the sutures followed by secondary skull deformities that follow a law that says “the normal bone growth is inhibited on the orthogonal direction relative to the closed suture; a compensatory bone growth develops in parallel with the closed suture”. Virchow’s law partly maintains its validity today. Virchow’s craniosynostose classification chart is still of reference today. However, the early closure of a suture may not always result in a compensatory bone growth. On this ground, the early closure of the sutures should not be defined by the secondary deformity but by the suture or the sutures that were affected (Virchow R., 1959).

Craniosynostoses are common malformations that occur in 1 out of 2000 live new-born (Shilito & Matson, 1968). The data in the literature show that scaphocephaly has an incidence of 0.4 out of 1000 new-born, it has male preponderance M/F = 3,5/1 and familial case occurrence – rare (Shilito & Matson, 1968).

Craniosynostoses are important for two particular reasons: their occurrence is a significant health problem. From a pathogenic point of view, they represent a model for the study of genetic and/or environmental factors causing malformations. The molecular basis of the most types of craniosynostoses is known today and the genetic tests result in an accurate diagnostic. The identification of the genetic lesions does not have a direct impact on the treatment of the patients with this kind of affections but allows an accurate prenatal diagnosis [**47**]. 

## Classification

Arseni et al., (1985) [*6] classifies the craniosynostoses in four groups:

- Simple – a single synostosed suture 

- Complete – two or more synostosed sutures

- Complex – the skull abnormality is included in a malformative complex

- Accompanying – the minor cranial dismorphy in minor and constitutes a side effect of other disorders – metabolic, hematological

The typical treatment in all craniosynostoses is surgery. The goal of the treatment is to reduce the intra-cranial pressure and to correct the deformities of the skull and face bones. This goal is achievable today by pre- and post-operatory 3D CT scan evaluation.

The aims of the surgical correction of these conditions are to counteract the aesthetic and functional anomalies of the craniofacial skeleton, to restore the normal spatial relationship between the skull and the contained neural structures. Also sometimes, it is required to correct the possibly associated abnormalities of the cerebral blood flow and cerebrospinal fluid (CSF) circulation. 

Among the various forms of abnormal cranial and facial bone development, those collectively defined as simple craniosynostoses allow the neurosurgeon to achieve all the previously mentioned therapeutic goals by means of relatively simple surgical procedures.

The definition of simple craniosynostosis however, implies further characteristics. Namely, the functional and anatomical anomalies should be easily identified based on the mere physical examination without the necessity of specific investigations. In other words, simple craniosynostoses tend to repeat their peculiar phenotype, which allows their recognition already at the first inspection. Furthermore, their natural history and prognosis can be predicted with good reliability. Consequently, the family can be offered a definite surgical plan, adequate information on the risks and the advantages of the surgical correction, as well as on the long-term outcome.

The most frequent simple craniosynostosis is scaphocephaly (the early closure of the sagittal suture). Therefore, the authors focus on this type of craniosynostosis in this article.

The complex craniosynostoses are completely different. The phenotypic recognition may remain uncertain in many cases, such as, for example, in Crouzon syndrome, Apert’s syndrome, Pfeiffer-Carpenter syndrome, cloverleaf skull [**48**], where the phenotypic appearance is just a continuum of apparently different clinical patterns actually depending on mutations of a single gene FGFR2 (clinical variability). On the other hand, different genes may express similar clinical forms, such as the Saethre-Chotzen syndrome, which may depend on mutations of both TWIST and FGFR3 genes (genetic heterogeneity). Furthermore, in these malformations, the clinical phenotype can remain under-expressed in the first months of life, see, for example, the Crouzon's syndrome that may firstly present as the simple fusion of sagittal or coronal suture.

## Material and method

The authors present 98 cases of scaphocephaly diagnosed and surgically treated in the Neurosurgery Department of “Dr. Bagdasar-Arseni” Emergency Hospital during 10 years (2000 – 2009).

**Fig. 1:**
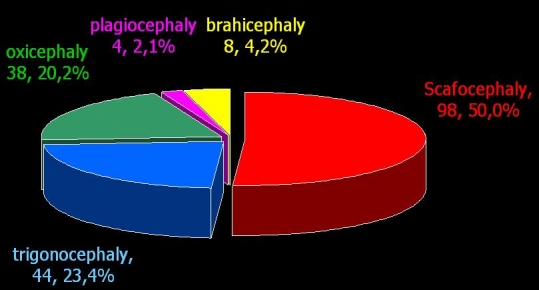


The total number of craniosynostoses in this time interval was of 188 cases, scaphocephaly being preponderant (**Fig. 1**).

The commonly used investigations are: - Cephalograms - Plain skull X-rays - CT scan - 3D CT scan - MRI - Radioisotope scan (dangerous and outdated) - Ultrasonic prenatal diagnosis

**Fig. 2:**
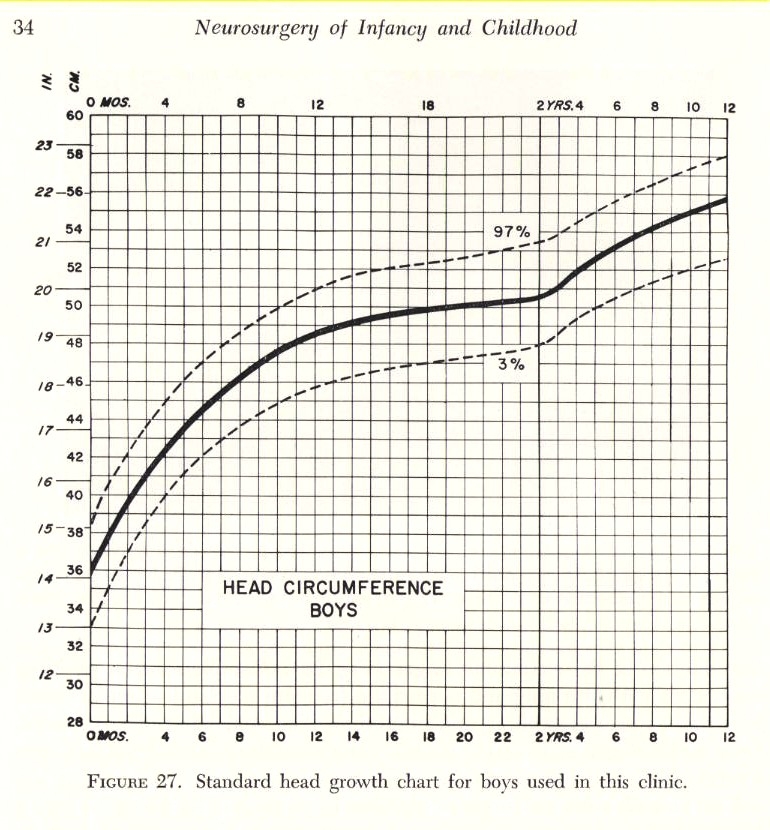


**Fig. 3:**
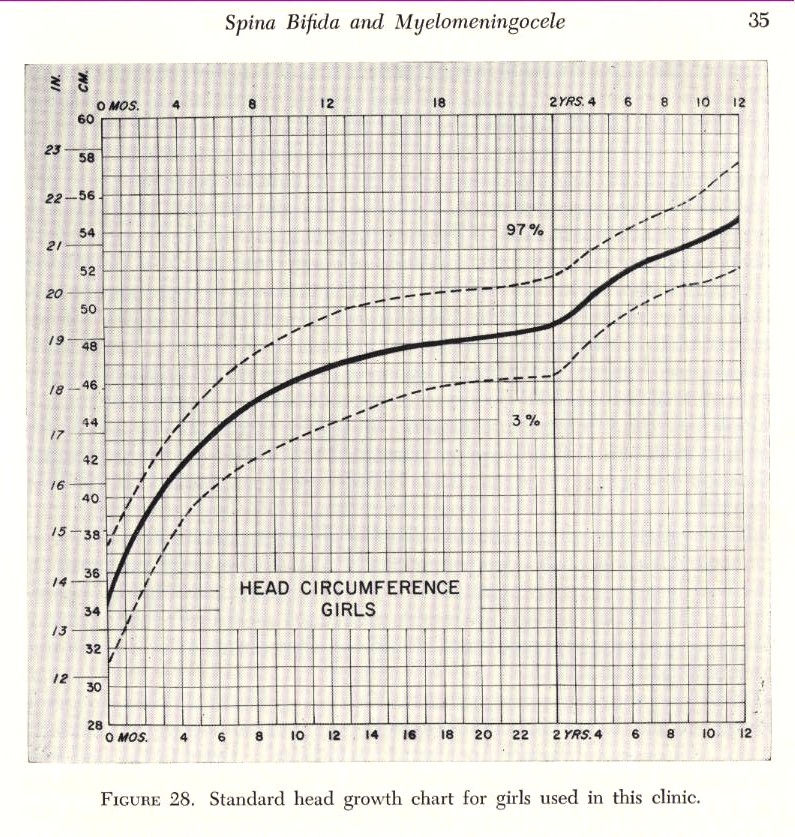


Certainly, the cranial circumference (cephalogram) is an important element in appraising the changes of the skull (**Fig. 2,Fig. 3**)

The deviations from the growth standard will immediately draw attention to the cranial abnormalities, hence, on the possible craniosynostosis disease.

The cephalic indices are altered, primarily to the ratio between the transversal and longitudinal diameter, which is less than 1.

**Fig. 4:**
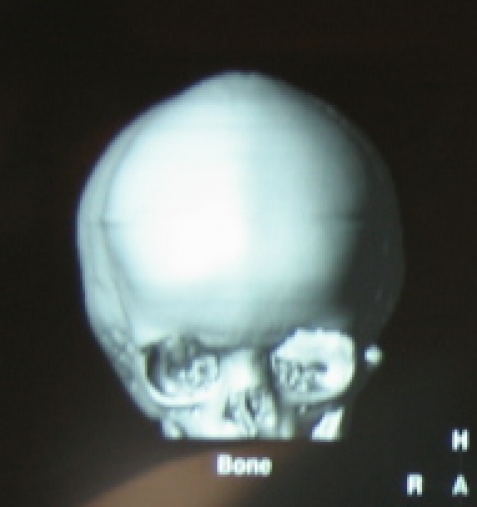
Frontal 3D CT in scaphocephaly

**Fig. 5:**
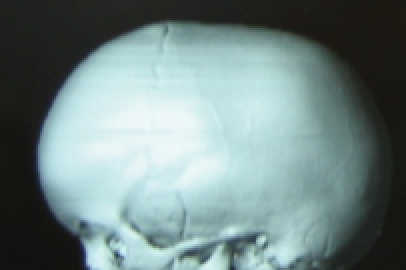
Lateral 3D CT in scaphocephaly

The 3D CT investigation is the preferred investigation. It yields rich information on the alteration of the normal skull shape and on the synostosed suture. It is most useful in planning the osteotomy and skull reshaping surgery (**Fig. 4,Fig. 5**).

**Fig. 6:**
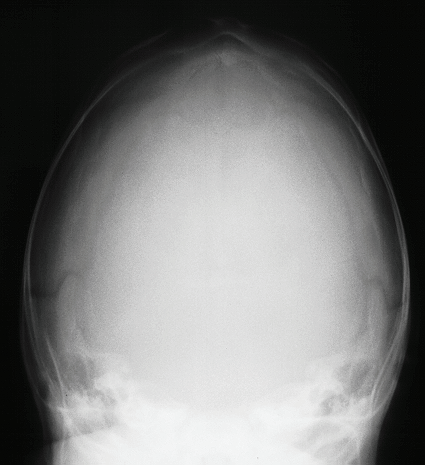
Skull X-ray in scaphocephaly AP imaging

**Fig. 7:**
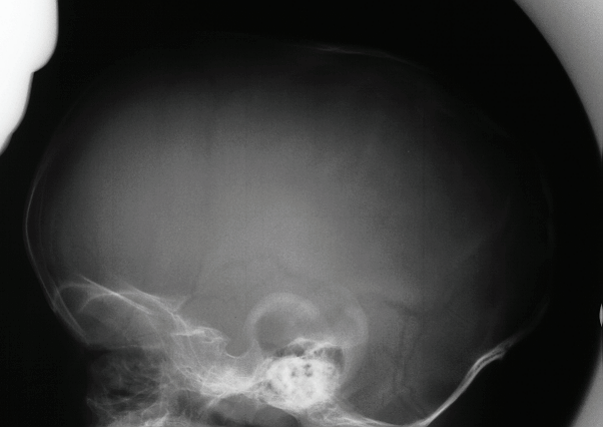
Skull X-ray in scaphocephaly lateral imaging

Certainly, the simple X-ray investigation maintains its diagnostic value but has the disadvantage of a supplementary child irradiation (**Fig. 6,Fig. 7**).

**Fig. 8:**
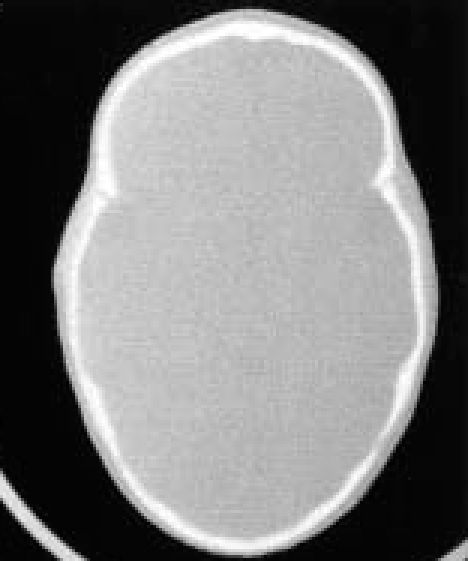
CT native bone window: pathognomonic aspect

The simple CT scan reveals the pathognomonic aspect of ‘boat-shaped’ skull and cranial shape in the transversal/longitudinal plane. In addition, native CT scan is useful in assessing ventricular size (**Fig. 8**)

The MRI is a very useful investigation in scaphocephaly by assessing the compressed neural and ventricular structures.

The surgical treatment is mandatory due to the following: • raised ICP • mental retardation • visual deficits/impairment • cosmetic aspect • skull deformity: psychosocial disturbance

The patient’s position during surgery: - Supine position: head flexed allows access to frontal and occipital area - Prone position: posterior two thirds of the sagittal suture.

**Fig. 9:**
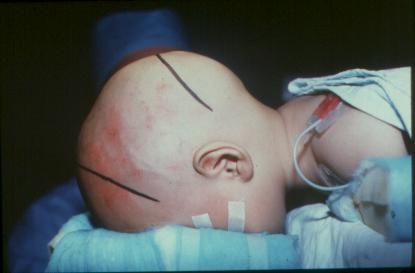
Patient’s position during surgery (prone position)

The prone position was preferred because it allows wide access to the anterior and posterior sagittal suture (**Fig. 9**).

The commonly surgical procedures used in scaphocephaly are: 

A. Simple linear craniectomy (2cm)

B. Extensive craniectomies (6-8cm)

C. Craniectomies & reconstructive procedures

The procedure of choice was extensive craniotomy (Stein & Schut, 1977) and reconstructive surgery facilitated by the development in craniotome and high speed drill tools.

**Figure:**
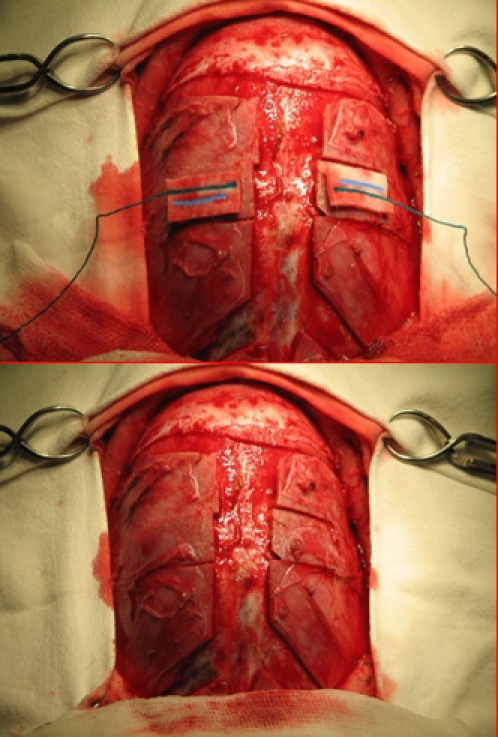
**Fig. 10 a, b.** Removal of the sagittal suture and lateral osteotomies to allow for brain growth

**Fig. 11:**
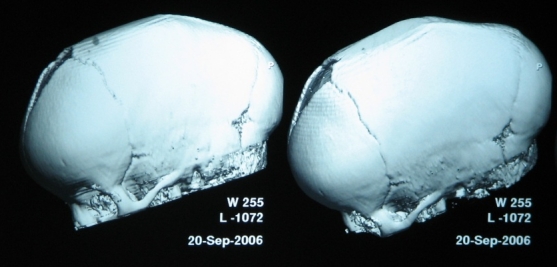
Preoperatory 3D CT in scaphocephaly

**Fig. 12:**
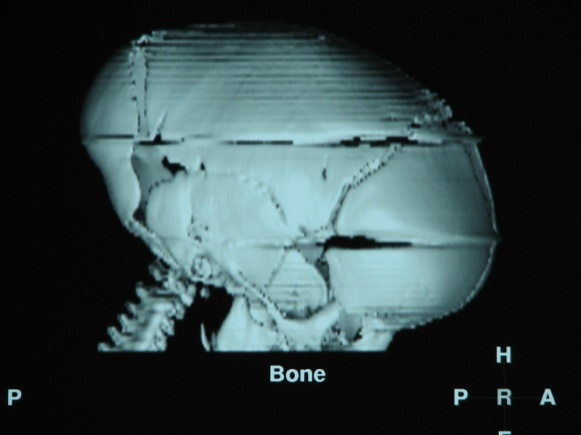
Postoperatory 3D CT in scaphocephaly

The pre- and post operatory 3D CT scan is the preferred investigation to assess the efficiency of craniotomies (**Fig. 11,Fig. 12**)

The post operatory complications appeared in 31 cases (31,6%)

– Seizures 11 cases 11,22%

– Neurologic transitory deficit 2 cases 2,04%

– Anemic syndrome 21 cases 21,42%

– Infection 2 cases 2,04%

– Hemorrhagic shock 1 cases 1,02%

– CSF fistula 1 case 1.02%

There was no post operatory death case. 

Postoperative management: 24 h in PICU 

The results of the surgery evaluated after 18 months were satisfactory in all cases from the neurologic, psychomotor development and cosmetic appearance. The exact evaluation is achieved by 3D CT scan (**Fig. 13 a,b**).

**Figure:**
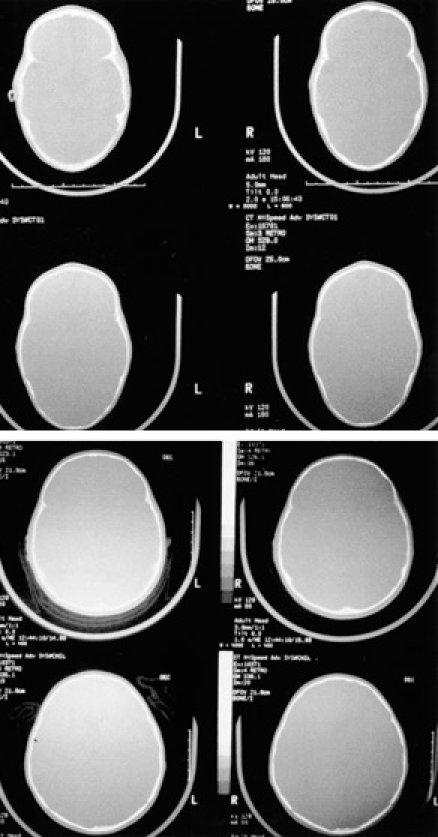
**Fig. 13 a, b.** 3D CT scan in scaphocephaly a – Preoperatory; b – Postoperatory

None of the cases required the reopening of the synostosed sagittal suture.

## Discussions

Scaphocephaly is a simple craniosynostosis caused by a precocious fusion of sagittal suture without other associated synostosis. Scaphocephaly is the most common isolated synostosis.

Compensatory skull growth produces uniform longitudinal elongation with frontal and occipital bossing and secondary head deformation.

The scaphocephaly incidence is of 56-58 %, Shilito & Matson (1968).

The data in the literature shows a general incidence of 0.4 out of 1000 new born with male preponderance M/F = 3,5/1 and rare familial cases Shilito & Matson (1968).

The clinical examination reveals some pathognomonic aspects: sagittal ridge, deformity apparent at birth. The clinical diagnostic is set in the first days/weeks of life. Often prominent forehead and disproportion is present (anterior region enlarged and parieto temporal region narrowed).

The skull expansion in the premature fusion of the sagittal suture is allowed by anterior fontanel and metopic suture. The general aspect of the skull is "boat-shaped" with narrow skull and cranial base and relatively normal facial development. 

Headache and vomiting are very uncommon clinical symptoms in small children.

Raised ICP can be observed only in toddlers. When the child is more than three years old, more symptoms and signs can be observed: headache, vomiting, seizures, visual impairment. No neurological deficit and no papilledema or optic atrophy was observed. In a few cases, mental retardation was present.

The following clinical symptoms require immediate surgical treatment in scaphocephaly: raised ICP, mental retardation, visual deficits/impairment, cosmetic aspect, skull deformity with psychological disturbances.

The authors note that the scaphocephaly surgical procedure requires a multidisciplinary team: neuroradiologist, craniofacial surgeon, pediatric neurosurgeon and pediatric anesthesiologist. The pediatric orthodontist is not involved, as this condition does not involve disorders in dental implantation.

Summarizing, the neurosurgical procedures in scaphocephaly are simple linear craniotomy (2cm), extensive craniotomies (6-8cm), craniotomies, and reconstructive procedures.

**Fig. 14:**
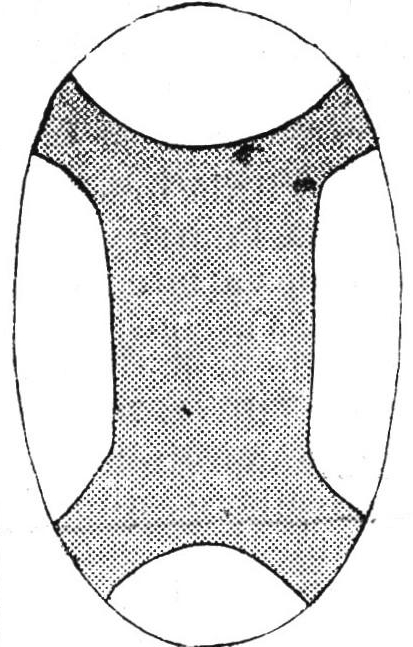
Stein&Schut (1977) procedure

The authors note that linear craniotomy was among the first procedures used in scaphocephaly treatment: linear simple sagittal craniotomy (Lanelongue, 1890), bilateral strip craniotomy (Ingraham, 1954). Subsequently extensive craniotomies (6-8cm) were the procedures of choice having the advantage of allowing brain growth. Examples of extensive craniotomies procedures are Raimondi procedure (1987), Venes & Sayers (1976), Stein & Schut (1977), Keyhole craniotomy Albright (1985). The Stein & Schut procedure consists of a large osteotomy of the sagittal suture continued with lateral anterior and posterior openings (**Fig. 14**)

The modern treatment of craniosynostoses involves craniotomies and reconstructive procedures allowing the skull to develop to a normal shape. The cerebral decompression to normal levels is achieved by applying surgery during the child’s first 6 months. The early procedure (during the child’s first 3 months) may ameliorate the child’s developmental delay.

Of the modern endoscopic procedures, we can mention the procedure presented by Jimenez et al (2004). The endoscope was used to guide the sagittal suture osteotomy in a 4-6 weeks scaphocephaly case. The osteotomy follows an anterior and posterior sagittal suture opening. The osteotomy was monitored subcutaneously by the endoscope. The procedure is minimally invasive but can cause lesions of the superior longitudinal sinus. The procedure is useful in the treatment of a 4-6 weeks old child.

The ultrasound prenatal diagnosis contributes to the early diagnosis of scaphocephaly and allows the planning of the surgical treatment during the child’s first 3 months of life.

## Conclusions

Scaphocephaly is the most frequent craniosynostosis and consists in the early closure of the sagittal suture. The clinic and imagistic diagnosis is straightforward in these cases. The neurosurgical decompression is mandatory during the child’s first 3-6 months of life. After that, the intra-operatory anemic syndrome aggravates the neurosurgical procedure. The treatment of a child with an abnormal head shape requires a team approach. The goal is to provide the most current diagnostic and treatment methods for the child, in a supportive environment. The team includes a neuroradiologist, craniofacial surgeon, pediatric neurosurgeon, and pediatric anesthesiologist.

The cranial remodeling allows unrestricted development of the brain and is facilitated by the 3D CT scan evaluation, by the development of the craniotome and high-speed drills, as well as of the modern fixation devices for barrel stave osteotomies.
